# Lightweight and High Impact Toughness PP/PET/POE Composite Foams Fabricated by In Situ Nanofibrillation and Microcellular Injection Molding

**DOI:** 10.3390/polym15010227

**Published:** 2023-01-01

**Authors:** Junwei Sun, Qian Li, Yufan Jiang, Jing Jiang, Lian Yang, Caiyi Jia, Feng Chen, Xiaofeng Wang

**Affiliations:** 1 School of Mechanics and Safety Engineering, Zhengzhou University, National Center for International Research of Micro-Nano Molding Technology, Zhengzhou 450001, China; 2 School of Materials Science & Engineering, Zhengzhou University, Zhengzhou 450001, China; 3 School of Mechanical & Power Engineering, Zhengzhou University, Zhengzhou 450001, China; 4 College of Material Science and Engineering, Zhejiang University of Technology, Hangzhou 310014, China

**Keywords:** polypropylene, in situ nanofibrils, microcellular injection foaming, cellular structure, impact strength

## Abstract

Polypropylene (PP) has become the most promising and candidate material for fabricating lightweight products. Microcellular injection molding (MIM) is a cost-effective technology for manufacturing porous plastic products. However, it is still challenging to fabricate high-performance PP microcellular components. Herein, we reported an efficient strategy to produce lightweight and high impact toughness foamed PP/polyethylene terephthalate (PET)/polyolefin-based elastomer (POE) components by combining in situ fibrillation (INF) and MIM technologies. First, the INF composite was prepared by integrating twin-screw compounding with melt spinning. SEM analysis showed PET nanofibrils with a diameter of 258 nm were achieved and distributed uniformly in the PP due to the POE’s inducing elaboration effect. Rheological and DSC analysis demonstrated PET nanofibrils pronouncedly improved PP’s viscoelasticity and crystal nucleation rate, respectively. Compared with PP foam, INF composite foam showed more stretched cells in the skin layer and refined spherical cells in the core layer. Due to the synergistic toughening effect of PET nanofibrils and POE elastic particles, the impact strength of INF composite foams was 295.3% higher than that of PP foam and 191.2% higher than that of melt-blended PP/PET foam. The results gathered in this study reveal potential applications for PP based INF composite foams in the manufacturing of lightweight automotive products with enhanced impact properties.

## 1. Introduction

Polypropylene (PP) is one of the most widely used thermoplastic all over the global plastic market. Owing to its advantageous performances, including low cost and density, good processability, excellent chemical and temperature resistance and high mechanical properties, PP has been widely used in many fields such as automotive, packing, construction and consumer electronics [1]. Using auto parts as an example, more than 20% of automotive interior and exterior are made from PP and PP-based composites [2]. In this context, PP becomes the most promising and candidate for fabricating lightweight and high-performance products. Driven by the economic and social interests, such as cost-saving, healthy and sustainable social development, fabricating lightweight and high-performance PP products shows a promising future for both academia and industry.

Material lightweighting, structural optimization design and manufacturing process innovation are three effective ways to achieve lightweight products [3]. Polymer foams are a kind of lightweight material made up of a polymer matrix with gas cells incorporated in it. The internal porous structure can also endow the polymer foams more unique functional properties such as thermal insulation, electromagnetic shielding, oil-adsorption and tissue engineering [4,5]. At present, there are three continuous fabrication process of porous polymeric materials, including extrusion microcellular foaming, 3D printing and microcellular injection molding (MIM). The extrusion foaming process provides higher productivity, ease of control and versatility in end-product properties and profiles, but it cannot fabricate foamed products with complex structures [6]. Three-dimensional printing has great potential for the preparation of porous materials. It is not constrained by existing manufacturing methods and can be tailored to operational applications and environmental conditions [7,8]. However, product dimensional accuracy, surface roughness and raw material limitations constrain its industrialization and large-scale application to a certain extent [9]. MIM, as a promising technology to fabricate foamed products, has been applying in many industries, due to its easy processing flexibility, good scalability, environmental benefit and high efficiency to mold products with complex structures [10]. However, there remains challenges to fabricate lightweight and high-performance PP foams by MIM owing to the following reasons. First, the PP molecular chain is prone to be disentangled due to its linear molecular chain structure, leading to its low melt strength and viscoelasticity [11]. Thus, cell collapse and coalescence usually happen at relative high foaming temperature in the MIM process, resulting in poor mechanical performance [12]. Second, unlike the batch foam in a static condition, MIM contains a complex behavior of fountain flow. Both foaming pressure and temperature vary during the whole filling process, resulting in different cell nucleation time and then different melt conditions for cell growth under different shearing history. Therefore, it is difficult to form a homogeneous and stable cell structure [13], and further deteriorates mechanical performance. Third, the mechanical properties of foamed products, especially stiffness or hardness, are generally inferior to that of solid samples [14,15]. It is due to the fact that the internal cells, which normally act as stress concentration points, may decrease the mechanical properties corresponding to the weight reduction [12].

Therefore, promoting the foaming ability of PP during MIM process, and further achieving uniform micro/nano-scale cells, plays an important role in enhancing mechanical properties. In situ fibrillation-based technology is increasingly recognized as an environmentally sustainable, inexpensive and high-throughput process for the fabrication of in situ nanofiber (INF) reinforced composites. INF composites are potential candidates to be substituted by carbon/glass fiber reinforced composites [16,17]. During the INF process, the fibrillar network, which is developed by physical entanglements without dispersion difficulty for the fibrils with high aspect ratios, is formed from well-dispersed phases [18]. The in situ deformation and orientation of the incorporation dispersed phase in the matrix can form micro/nanoscale fibrils and disordered physical network offering unique advantages, including the enhancement of melt viscoelasticity, crystallization, mechanical properties and foamability [19,20]. Many studies have indicated that the lightweight and high-strength polymer foams can be prepared by combining in situ fibrillation technology with microcellular foaming process [21,22].

The incompatibility between the matrix and the dispersed phase is beneficial for the formation of physical fibrillar network. While on the other hand, the weak interfacial interaction originating from the incompatibility makes the micro/nanoscale fibrils strip out of the matrix at the interface due to the stress concentration [23]. Therefore, the incorporation of compatibilizer is necessary to regulate the contradiction between in situ fibrillation and interfacial adhesion on compatibility requirements. Polyolefin-based elastomer (POE), one of the most prominent thermoplastic elastomers, has a unique combination of flexibility, good processability and elasticity due to its micro-phase separation structure [24]. It is well known that POE has been widely using as a toughing agent for polymer composites. Recently, a new concept of compatibilization using thermoplastic elastomers as interfacial agents for immiscible polymer blends has been proposed [25,26,27]. A few studies have reported that the hardness and toughness of composites were synergistically enhanced by interfacial compatibilization and toughening after introducing thermoplastic elastomers [28,29]. Nevertheless, to our best knowledge, there is little research on the influence of POE on INF composite fabrication and corresponding microcellular foaming behaviors.

In this study, INF technology associated with the MIM process was applied to fabricate lightweight and excellent toughness INF composite foams. First, the PP/PET/POE composites with PET nanofibrils were prepared by coupling twin-screw compounding with melt spinning. Afterwards, the phase morphology, rheological properties, and isothermal crystallization behaviors of prepared the PP-based composites were analyzed. Then, MIM experiments were carried out to fabricate PP foams, PP/nanofibrous PET foams, and PP/nanofibrous PET/POE foams. PP/spherical PET and PP/spherical PET/POE foams were also prepared for comparison with the same process setting. The cellular morphologies of all foams, including parallel and perpendicular to flow direction, were investigated to identify the role of PET nanofibrils and POE particles in improving PP’s foaming ability. Finally, impact testing was performed to evaluate the mechanical performance of PP and PP-based composite foams. The synergistic toughening mechanism of MIM products by PET nanofibrils and POE were further explored.

## 2. Experimental

### 2.1. Materials

An isotactic PP (homopolymer, Moplen Z30S) was supplied as matrix polymer by Maoming Petrochemical Co., Ltd., with a density of 0.9 g/cm^3^ and a melt flow rate (MFR) of 25 g/10 min (230 °C/ 2.16 kg). The dispersed phase polymer employed in this work was PET (FC510) by Yizheng Chemical Co., Ltd. (Guangdong, China). The melt point of PP and PET was determined by DSC as 167°C and 253°C, respectively. Polyolefin elastomer (POE, LC565) was provided by Dow Chemical Investment Co. (Shanghai, China), with an MFI of 5 g/10 min at 190 °C/2.16 kg. The physical blowing agent, CO_2_ (99% purity), was obtained from Ningbo Wanli Gas Corporation (Ningbo, China). 

### 2.2. Preparation of Nanofibrillar PP based Composites and Foams

**Figure 1:**
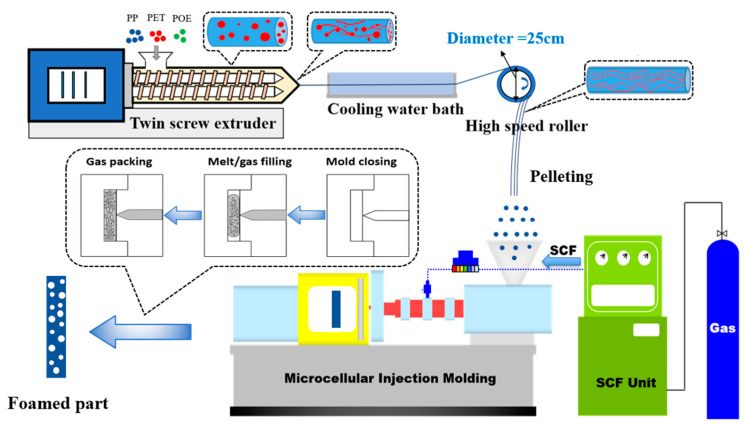
Schematic fabricating of INF composites and foams.

The in situ nanofibril (INF) process combining twin-screw compounding with high-speed hot drawing is presented in this study, and is shown in Figure 1. At first, PP pellets were dried at 80 °C for 6 h, and PET pellets were dried at 130 °C for 8 h to avoid hydrolysis during compounding. A co-rotating twin-screw extruder (TSE, Ruiya Co., Ltd., Foshan, China), with a screw diameter of 22 mm and an aspect ratio of 40, was used to compound PP, PET and POE pellets. In order to compare the PP based binary blends and triple blends, the dosage of PP matrix was maintained at 90 wt.%. The formulations of neat PP and PP based composites are listed in Table 1.

The processing temperature of extruder barrel was set to 7 segments from feeding zone to die, specifically 160 °C, 190 °C, 230 °C, 265 °C, 265 °C, 265 °C and 255 °C. A screw speed of 80 rpm was used and feeding speed was kept at about 7.5 kg per hour. The extrudate from the die was connected with a custom-built fiber spinning system, comprised of a variable speed motor and a high-speed roller with diameter of 25 mm. The rotational motion of the roller drew the extruder, and the spherical PET domains was deformed into nanofibrils uniformly dispersed in PP matrix. By maintaining the rotational rate of the roller, the extrudate draw ratio was kept at 12.5:1. For comparison, an undrawn sample which only experienced traditional melt blending was also studied. The neat PP went through the same process to ensure the same thermal history. It should be mentioned that many trials prior to the formal experiments have been performed to acquire the above processing parameters.

The pelletized INF composites were used as raw materials for fabricating fibrillary PET reinforced PP composite foams by a 120-ton injection molding machine (FE120-430h, YIZUMI, Guangdong, China), equipped with a Mucell SCF delivery system (T-100, Trexel Inc., Wilmington, MA, USA). MIM experiments were conducted to prepare PP based composite foams. The main processing parameters, including melt temperature, mold temperature, injection speed and gas dosage were set to be 190 °C, 35 °C, 75 mm/s and 0.7 wt.%, respectively. The shot size adjusted to maintain the resulting weight reduction was 20% compared with the solid sample. For comparison purposes, neat PP and PP based melt-blended composite foams were also prepared under the same conditions.

### 2.3. Characterization

#### 2.3.1. INF Composites and Foams Morphology Characterization

A scanning electron microscope (SEM, Tuscan Mira LMS, Czech) was used to observe the sample’s micromorphology. All pelletized materials were first compressed into a rectangular plate at 190 °C under 10 MPa by a vacuum hot compression machine (K14L20VHE), and then were cryogenically fractured in liquid nitrogen. The fracture surface of the sample was coated with a thin layer of platinum to avoid electron charging during SEM observation. In order to observe the variation of PET domains, all composite samples were exposed to xylene vapors for 1.5 h at 140 °C, followed by ultrasonic cleaning in deionized water twice to remove the PP matrix. With the obtained micrographs, Image J software (version 1.8.0.) was used to define the spherical and fibril PET domain size.

**Figure 2:**
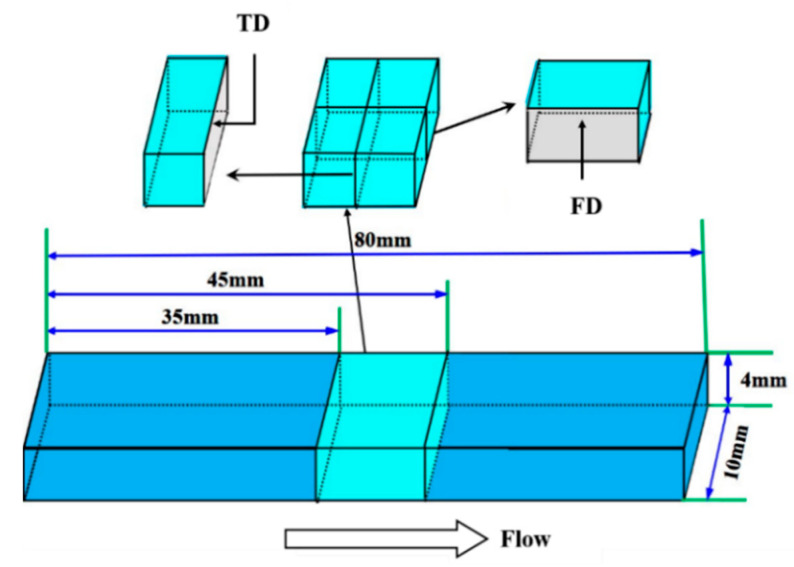
The geometry of impact specimen and characterization areas. FD, flow direction; TD, thickness direction.

In order to observe the cellular morphology inside the foamed sample, the impact spline was cooled in liquid nitrogen for 30 min and immediately fractured along melt flow direction (FD) and thickness direction (TD) to obtain the real cellular morphology. The geometry of the impact sample and SEM characterization regions are illustrated in Figure 2. Finally, the fracture surface was coated with platinum layer by a sputter coater (Quorum SC7620). Image J software was further used to collect the quantitative information of cells.

The average diameter of the cells can be calculated by the following formula:

(1) $$D=\frac{\sum_{i=1}^{n}d_{i}}{n}$$

where $d_{i}$ is the diameter of a single cell, *n* is the number of cells.

The population density of the cells can be calculated using the following equation:

(2) $$N={\left(\frac{n\times M^{2}}{A}\right)}^{\frac{3}{2}}\;\;$$

where *M* is the magnification of the SEM microscope, and *A* is the area of the image.

**Figure 3:**
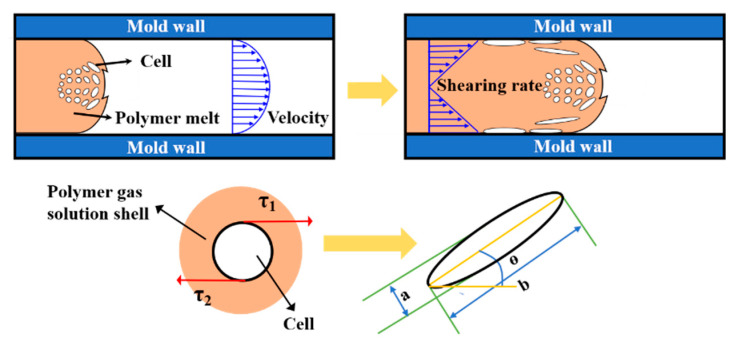
Schematic diagram of the length-diameter ratio and cellular offset angle.

As in conventional injection molding, a typical three-layer structure is obtained with the increase in distance from the mold wall during the MIM process [30]. In order to investigate the variation of stretched cells consisted in the shear layer, the length–diameter ratio of the cells and the offset angle of the cells were used to characterize quantitatively the deformation of cells. As can be seen from Figure 3, with the increase of length–diameter ratio of the cells, the offset angle deflection flow direction became smaller, indicating a bigger cell deformation. The ratio of length to diameter (*d*) was calculated by the following equation [31]:

(3) $$d=\frac{b}{a}$$

#### 2.3.2. Rheology Characterization

A rotational rheometer (DHR-2, TA, USA) was used to investigate the rheology behavior of the PP and PP based composites. A dynamic strain sweep was conducted first to identify the linear viscoelastic region. Afterwards, the dynamic frequency sweep tests were applied with the frequency (*ω*) ranging from 0.01 to 100 rad/s at 190 °C to obtain storage modulus (*G’*), loss factor (*tan δ*) and complex viscosity (η*). All tests were conducted under the protection nitrogen atmosphere to avoid oxidative degradation of melt.

#### 2.3.3. Isothermal Crystallization Characterization

A differential scanning calorimetry (DSC, Q2000, TA, New Castle, DE, USA) was used to investigate the isothermal crystallization behavior. The samples were heated from 40 °C to 200 °C at a 10 °C/min heating rate and held for 5 min to eliminate thermal history. Subsequently, the samples were cooled at 10 °C/min to 130 °C and held for 20 min. All measurements were performed under nitrogen atmosphere protection.

#### 2.3.4. Mechanical Characterization

Izod impact testing was conducted by using an impact tester (PTM7000, SANS, Shenzhen, China) according to ISO 180. Before testing, all samples were dried at 60 °C in a vacuum for at least 3 days to remove any moisture. All specimens were notched in the middle with a depth of 2 mm using a standard cutter, and then placed horizontally with the notch oriented away from the pendulum and broken by a hammer with an energy of 2 J. At least five specimens were tested for each test to evaluate the reliability and stability of results.

## 3. Results and Discussion

### 3.1. Composite Morphology

**Figure 4:**
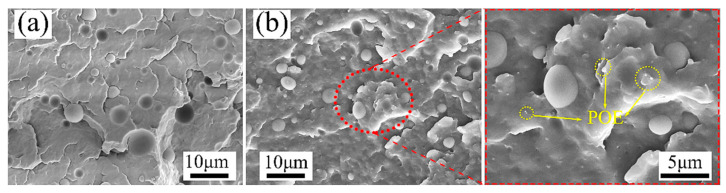
SEM images of the melt-blended composites cryogenic fractured surfaces: (**a**) PP/PET(S), (**b**) PP/PET/POE(S).

For polymer composites, the phase morphology and distribution of dispersed domains play an important part in determining their foaming behavior and mechanical performances. Figure 4 shows the phase morphology of melt-blended PP/PET blends with and without POE particles. It can be found that both micrographs exhibit no evidence of fibrillation and typical sea-island structure with a homogeneous dispersion of the minor blend component as spherical particles. In the PP/PET(S)/POE composite, the phase interface of PP matrix and PET domains becomes rougher compared to the PP/PET(S) blend, indicating the interfacial adhesion is improved. In other words, the incorporation of POE particles plays a role like compatibilizer, which reduce the interfacial energy and facilitates the stress transfer across the matrix–particle interface [32]. Furthermore, after 3 wt.% content of POE is added (Figure 4b), no agglomeration phenomenon is detected. The average diameter of PET particles is reduced from 4.3 μm to 2.9 μm, and the POE particles are uniformly distributed in PP matrix as yellow dotted circles marked (Supporting Information Figure S1).

**Figure 5:**
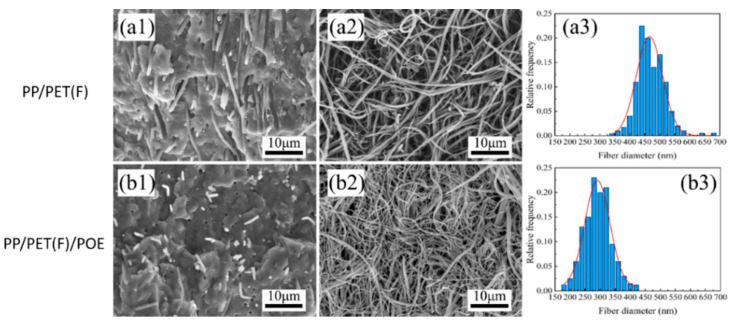
SEM images of fibrillary PP based composite before (**a1**,**b1**) and after etching PP matrix (**a2**,**b2**) with xylene vapor and size distribution of PET fibrils (**a3**,**b3**).

Figure 5 presents the morphology of INF composites before and after etching PP matrix using xylene. In Figure 5(a1,b1), it was found that the PET domains have been fibrillated into fibrils and uniformly distributed in PP matrix due to the combined shearing and stretching effect during the INF process. Moreover, the addition of POE particles significantly reduces the degree of PET fibrils shedding from the PP matrix, which indicates that the interfacial adhesion between PP and PET fibrils are improved due to the interface compatibilization of POE incorporation [33]. Figure 5(a2,a3) and Figure 5(b2,b3) obviously depict that all fibrillated PET fibrils with a big length–diameter ratio formed an entangled network structure. Compared to PP/PET(F) composite, the average diameter of PET fibrils was decreased from 465 nm to 258 nm, and the fibril distribution is more uniform after adding POE. The effect of POE on the PET fibrils morphology agrees with that of PET spherical domains discussed above. These results reveal that a small amount of POE particles are beneficial to obtain an entangled nanofibril PET network with nano-scale diameter and uniform distribution, which can effectively promote crystallization and enhance melt viscoelasticity, and thus greatly improve the foamability [34].

### 3.2. Rheological Behavior

**Figure 6:**
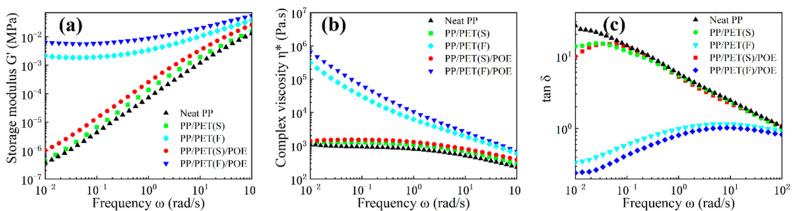
Rheological properties of neat PP, melt-blended and INF composites: (**a**) storage modulus (G’), (**b**) complex viscosity (η*) and (**c**) loss tangent (tan δ).

As rheological behavior has a significant influence on the foaming behavior of polymers, dynamic frequency sweep tests were carried out to characterize the effect of PET fibrils and POE particles on the linear viscoelastic responses of the PP matrix. Figure 6 shows the plots of storage modulus (*G*’), complex viscosity (η*) and loss tangent (tan δ) as a function of frequency. It is observed from Figure 6a that the slope of the *G*’ curve for neat PP, PP/PET(S) and PP/PET(S)/POE was about 2, indicating the rheological behavior of a melt with liquid-like properties [35]. PP/PET(S) and PP/PET(S)/POE exhibited a higher *G*’ curve than that of neat PP, especially in the region of low frequencies. We attributed this shift in *G*’ to the presence of the solid spherical PET domains that behave as rigid and influence the stress relaxation behavior of the PP matrix [36,37,38]. Remarkably, INF composites displayed an increment of *G*’ and a plateau occurred at low frequency, owing to the constraint formed by the PET fibril network as well as the increased entanglements between PET fibrils and PP molecular chains [39,40]. Besides, *G*’ of PP/PET(F)/POE was bigger than that of PP/PET(F), particularly at low frequencies, mainly attributing to the higher fibril aspect ratio and increased molecular entanglements caused by the spatially linked structure of PET nanofibrils [40].

As Figure 6b illustrates, both INF composites presented a more obvious shear shinning effect and typical non-Newtonian fluid behavior relative to the other materials. It was also detected that INF composites showed much higher η* over the frequency range, especially at low frequency, than that of neat PP and melt-blended composites. The enhanced η* is attributed to the fact that the PET fibril network, acting as the skeleton within the PP matrix, obviously restricts the motion of PP molecular chains. Under higher frequency, the decline in the value of η* was found to be more significant for INF composites, which is attributed to the higher orientation of PET nanofibrils, and their restrictions to the motion of PP molecular chains become weaker. Figure 6c shows the variation of tan δ with different shear frequencies, aiming to explore the influence of morphological variation of PET domains on the elastic response of PP matrix. Both neat PP and melt-blended composites exhibited a typical characteristic of viscoelastic liquids, with tan δ decreasing completely with an increment in frequency, and the values of tan δ bigger than 1 in the certain frequency region. These results match the Winter–Chambon criteria properly [41]. In contrast, the INF composites showed a slow increase in tan δ with increasing *ω*, and the values of tan δ were less than 1. In addition, the tan δ nearly expressed independence of *ω* under the high frequency regions. This indicates that the PET-nanofibril network structures formed in the PP matrix, which can sharply reduce viscous dissipation and contribute to the transition of a liquid-like behavior to a solid-like behavior. Similar results have been obtained from previous studies [42].

### 3.3. Crystallization Behavior

**Figure 7:**
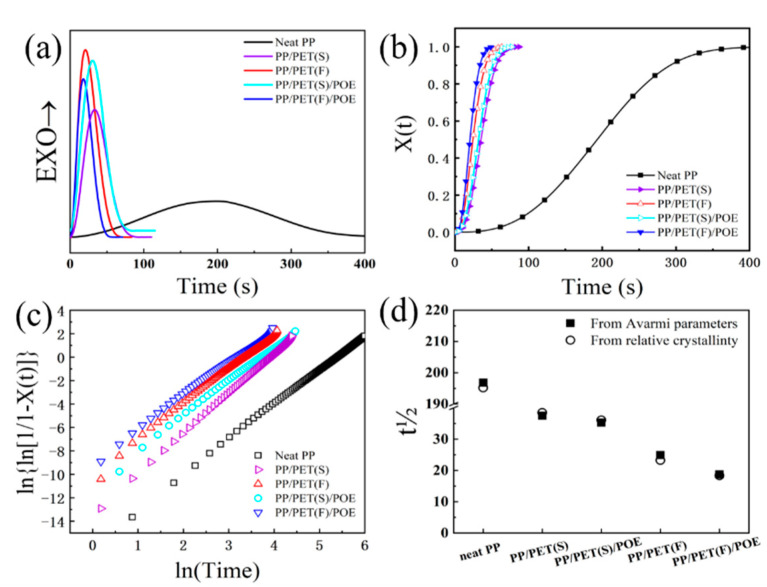
Isothermal crystallization behavior of neat PP, melt-blended and INF composites at 130 °C: (**a**) Isothermal crystallization exotherms as a function of time; (**b**) Relative crystallinity, X(t), as a function of time; (**c**) Isothermal crystallization kinetics analyzed curves; (**d**) Half-time, t1/2, of crystallization determined from the Avrami analysis and the relative crystallinity shown in (**b**).

Aside with rheological behavior, crystallization is another important factor in determining a composite’s foamability. The heat flow traces during isothermal crystallization at 130 °C for both neat PP and PP-based composites are displayed in Figure 7a. All heat flow curves show a single peak as is typical for isothermal crystallization of semi-crystalline polymer. Additionally, the extreme wide crystallization peak of neat PP is observed, indicating a rather slow crystallization behavior due to the low mobility of molecular chains [43]. Compared with neat PP, INF and melt-blended composites exhibit much narrower and sharper crystallization peaks. The addition of spherical or fibril PET domains accelerates the isothermal crystallization characteristic because of the nucleation effect of heterogeneous crystallization. Figure 7b shows the relative crystallinity *X*(t) as a function of time for all samples. As seen, all curves have a similar sigmoidal shape. PP crystallization characteristic was mostly enhanced for INF composites. Compared with melt-blended composites, PET nanofibrils in INF composite have much bigger specific surface areas than that of spherical PET domains, which thereby can promote the crystallization rate as function of nucleation agents. Besides, previous studies have indicated that PET nanofibrils incorporation could also facilitate the PP molecular mobility effectively [44], and hence enhance its crystallization characteristic. Furthermore, the fastest crystallization rate is obtained for the PP/PET(F)/POE composite due to the synergistic nucleation effect of PET nanofibrils and POE fillers.

A thermodynamic analysis of isothermal crystallization based on the Avrami model was conducted at 130 °C to further evaluate the influence of PET nanofibrils on crystallization. The Avrami plots are shown in Figure 7c, and corresponding Avrami parameters are also summarized in Table 2. The Avrami *n* exponent of all samples varied between 2–3. Although the heterogeneous crystal nucleating effect of the additive could generally promote spherulitic crystal growth resulting in the increase of *n* value beyond 3 [45], the trans-crystallization along the nanofibers could promote a two-dimensional disk shape and one-dimensional rod-like crystallization which correspond to *n* values between 2–3. It is also of note that PP/PET(F)/POE shows a significant three-magnitude and one-magnitude higher *Z*_t_ value than that of neat PP and PP/PET(F), respectively. It is well known that polymer crystallization contains crystal nucleation and growth. It is necessary to note that the existence of PET nanofibrils can promote the crystal nucleation rate as explained above. However, the crystal nuclei are normally subject to high resistance during the growth owing to the big viscoelastic property. It is difficult to compare the overall crystallization rate directly from the values of *Z_t_* because the unit of *Z_t_* is s^-n^ and *n* is not constant for the samples. Therefore, the half-time of crystallization (*t*_1/2_, the time required to achieve 50% of the final crystallization of the samples) is computed for the purpose of discussing the kinetics of crystallization [44]. Figure 7d illustrates the value of *t_1/2_* as a function of materials. The experimentally determined *t*_1/2_ from the relative crystallinity data was plotted alongside the *t*_1/2_ values derived from Avarima equation. It can be clearly distinguished from Table 1 that the *t_1/2_* of INF composites took dozens of seconds and was much shorter than that of neat PP. Intriguingly, compared with PP/PET(F), PP/PET(F)/POE had a shorter *t_1/2_*. We attributed this result to the smaller diameter and larger aspect ratio of PET nanofibrils caused by adding 3 wt.% POE, which offered a larger surface-to-volume ratio for crystal nucleation. Therefore, the gathered results give rise to the suggestion that the synergistic effect of PET nanofibrils and POE particles substantially enhance the PP matrix crystallization kinetics thermodynamically.

### 3.4. Cellular Morphology

**Figure 8:**
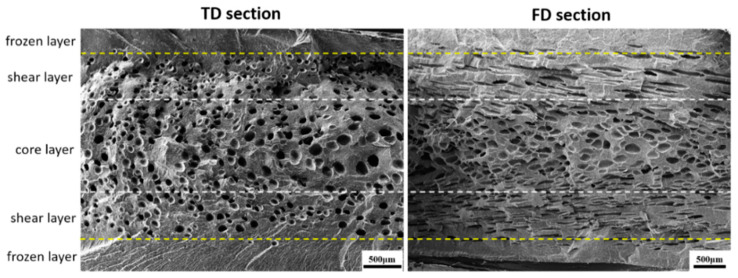
Integral section structure of microcellular PP/PET(F)/POE samples.

It is known that the differences of shearing stress and temperature along TD and FD usually generate a nonuniform cell structure, which deteriorates the mechanical properties of the foamed parts. An RMIM experiment was performed to fabricate neat PP and PP-based composite foams. Figure 8 shows the TD and FD SEM images of PP/PET(F)/POE composite foam. It is found that both integral section structures were quite consistent with the typical sandwich structure of microcellular injection molded part. No foam exists at the frozen layer touched with the mold surface. In fact, the frozen layer consists of two regions during filling process. The stretched cells are moved from the flow front to the mold surface and destroyed because of the big temperature gradient, and then a thin frozen layer is formed. The melt pressure increases gradually from the flow front to the gate, and the stretched cells are redissolved into the polymer melt under critical pressure. Then, a thicker solid-like layer is subsequently formed [46]. The shear layer contains stretched cells that are parallel to the flow direction. The stretched cells are pushed out from the flow front and cannot dissolve into the melt due to the unsaturated pressure. In the core layer, numerous spherical cells form after the filling process and are located in the central portion of the samples.

#### 3.4.1. Cellular Structures along TD

**Figure 9:**
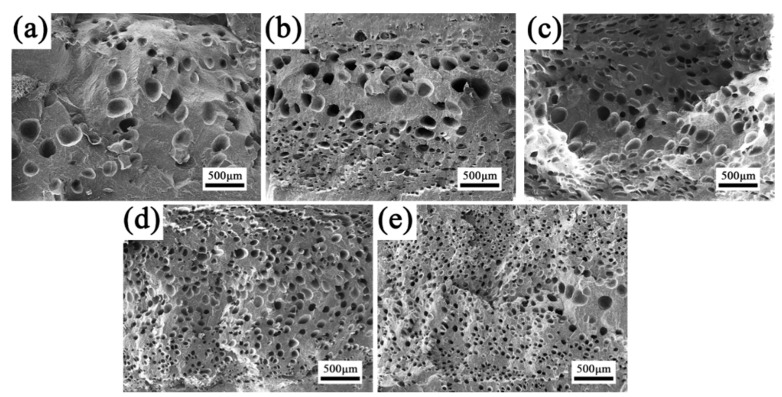
SEM images of the cellular structure in TD of foamed samples: (**a**) neat PP, (**b**) PP/PET(S), (**c**) PP/PET(S)/POE, (**d**) PP/PET(F), (**e**) PP/PET(F)/POE.

To investigate the effect of PET dispersed phase morphology evolution on the foaming behavior of PP, Figure 9 shows the SEM images of all foamed internal structures of the TD. It can be observed that the neat PP foam (Figure 9a) had a rather non-uniform cellular structure. Few large and irregular cells were found in the core layer of the foamed part. During RMIM, foaming always happens at relatively high temperature, at which the PP melt has rather low melt strength, leading to serious cell collapse and coalescence. For PP/PET(S) and PP/PET(S)/POE composite foams (Figure 9b,c), a finer cellular structure with smaller cell size and increased cell density was achieved around the core layer due to the improved viscoelasticity of melt and heterogeneous nucleation effect of PET and POE particles [31]. Moreover, many small sized and oriented cells appeared in the shear layer. The strong shear combined with foaming during the filling stage of MIM promotes cell collapse and coalescence, resulting in oriented cellular structure [47]. As shown in Figure 9d,e, obviously improved cellular structure characterized by reduced cell size, increased cell density and uniform cell distribution was achieved in INF composite foams. The refined cell structure of INF composite foams can be explained by two reasons. First, PET nanofibrils can enhance the heterogeneous nucleation efficiency at interfaces, which improves cell nucleation. Second, PET fibrils can not only promote the viscoelasticity and strain hardening behavior of PP [48], but also increase the crystallization rate, which in turn improves the melt strength of PP in relatively high temperature. Both reasons can contribute to the reduction of cell coalescences and collapses.

**Figure 10:**
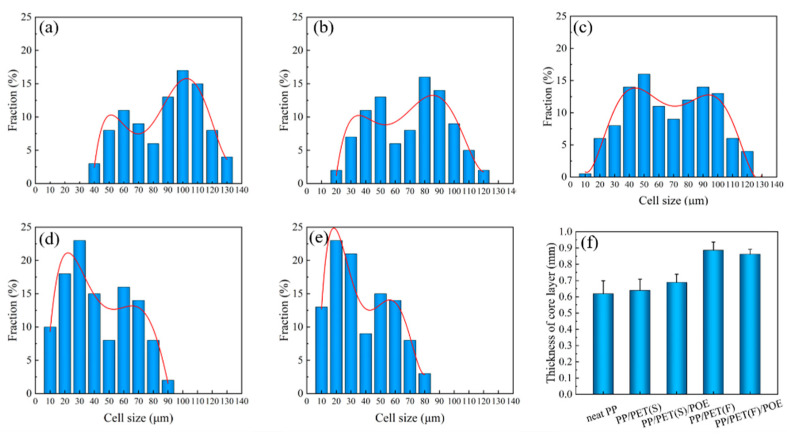
Cell size distribution of vertical sections: (**a**) neat PP, (**b**) PP/PET(S), (**c**) PP/PET(S)/POE, (**d**) PP/PET(F), (**e**) PP/PET(F)/POE; (**f**) thickness of core layer.

To quantitatively characterize the cell distribution, the cell size distribution of foamed samples was plotted in Figure 10(a–e). It is easy to note that all samples exhibit a bimodal cell structure, corresponding to the non-uniformity distribution of cells across the TD section. Noteworthily, the cell size in the core layer and shear layer were ranged 85–120 μm and 30–50 μm, respectively, for neat PP. After introducing PET nanofibrils, the corresponding cell size ranged to 60–80 μm and 10–40 μm, indicating the cell uniformity was greatly improved. It is well known that the uniformity of cell distribution is closely related to the phase morphology evolution of sandwich microstructure in RMIM sample. Figure 10f shows the thickness variation of core layer. It is found that the core layer thickness of INF composite foam was increased by approximately 45% compared to neat PP foam. During the filling stage of RMIM, the local shear viscosity of polymer melts with PET nanofibrils is increased and subsequently the flowability is reduced. Previous studies have indicated that the shear stress distribution in the TD section are more uniform, demonstrating the increment of core layer thickness [49].

Table 3 lists the statistical results of cell size and cell density of all foams at different sandwich structures. In both the core layer and shear layer, the cell size reduced while the cell density increased gradually, with the dispersed PET phase transferring from spherical domains to nanofibrils. The minimum cell size and maximum cell density of PP/PET(F)/POE foams in the core layer are about 50 μm and 1.6 × 10^7^ cells/cm^3^, respectively, which was greatly improved compared with that of neat PP. This indicates that the PET nanofibrils can homogenize the cell distribution, which was in accordance with the crystallization and cell morphology results discussed above.

#### 3.4.2. Cellular Structure along FD

**Figure 11:**
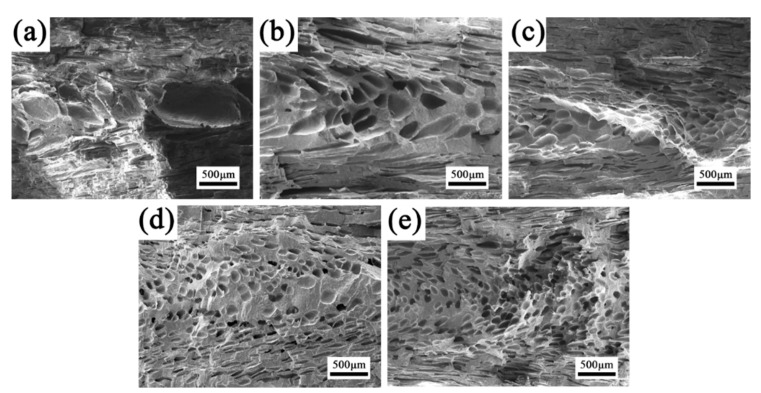
SEM images of the cellular structure along FD of foamed samples: (**a**) neat PP, (**b**) PP/PET(S), (**c**) PP/PET(S)/POE, (**d**) PP/PET(F), (**e**) PP/PET(F)/POE.

The cell morphologies parallel to FD are shown in Figure 11 to get a comprehensive understanding of the cell structure evolution of RMIM parts. It is noteworthy that the varied cell morphologies in the core layer of FD for all foamed samples are similar to that of TD as discussed above. However, many deformed cells were detected in shear layer due to the characteristic of shear flow of polymer melt in cavity. It can be concluded that most of the cells are extremely stretched and sheared during cell nucleation and growth in the filling stage. Furthermore, the cell deformation is obviously different among all PP-based composite foams.

**Figure 12:**
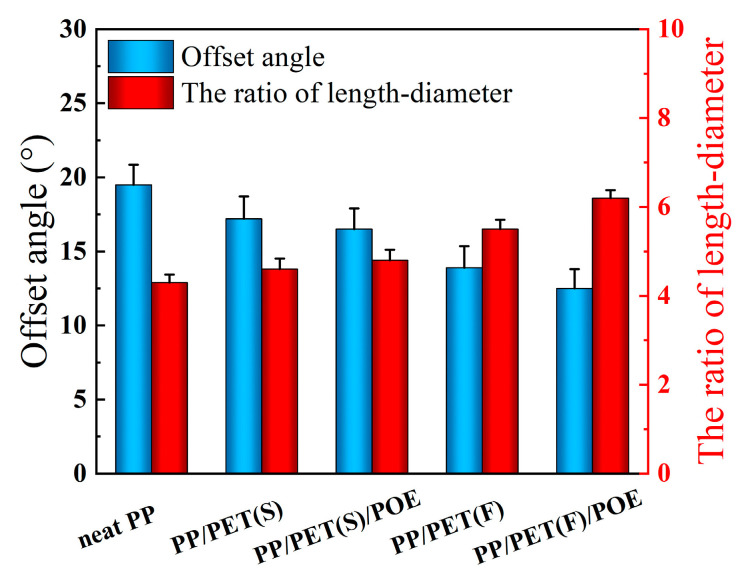
Cellular offset angles and length–diameter ratios of cells in shear layer along FD for different foamed samples.

Figure 12 shows the quantitative test results of cell deformation according to SEM micrographs. The ratio of length–diameter (*d*) is affected by the cell deformation, and normally higher cell deformation corresponds to a larger *d* value. The offset angle (*θ*) is affected by the melt temperature and local melt viscosity, and higher temperature and lower viscosities means a larger *θ*. It is clear that when compared with neat PP, the *d* of INF composite foams is larger, and the *θ* become smaller. This demonstrated that a lot of cells have occurred larger deformation. The main reason maybe that the presence of PET nanofibrils increase the resistance to polymer molecular chain motion, resulting in an increase in local viscosity and enhanced shearing stress. At the same time, the cell wall thickness decreased, and the ability of the cells to undergo deformation was reduced, leading to an increase in cell deformation. In particular, the biggest *d* and smallest *θ* were obtained for PP/PET(F)/POE foams. Besides the effect of PET nanofibrils, the POE with long chain branching can also improve the resistance of molecular chain motion [50].

### 3.5. Impact Properties

**Figure 13:**
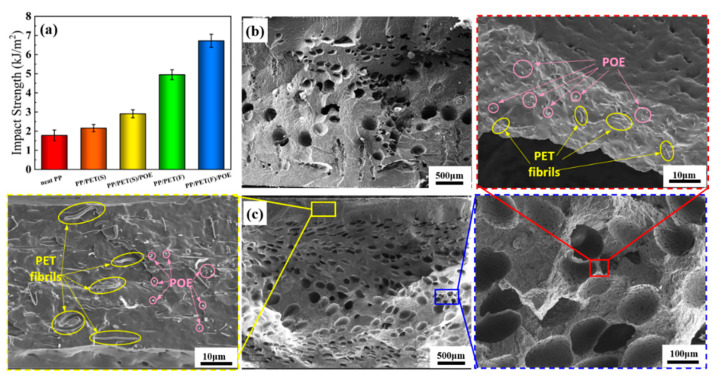
Impact test results: (**a**) Izod impact strength; (**b**) and (**c**) are the impact fracture surface morphology of neat PP and PP/PET(F)/POE foam, respectively.

Izod impact testing was conducted to evaluate the ductility of the injection molded foams at a relatively high deformation rate. Figure 13a plots the Izod impact strength of foamed samples. Obviously, the PP/PET(F)/POE foams exhibited the highest impact strength of 6.72 kJ/m^2^, increasing by 295.3% compared with the neat PP foams, and followed by PP/PET(S), PP/PET(S)/POE and PP/PET(F), which were increased by 191.2%, 71.2% and 27.1%, respectively. The sharply improved impact strength should definitely be correlated with the synergistic effect of cell structure and filler system (PET fibrils and POE additives). For one thing, the refined crystals caused by PET fibrils can promote the flexibility and toughness of PP matrix. Due to the weak cohesive forces between PET molecular chains, the PET fibrils can enhance the sliding ability of PP crystals [48]. Previous studies have demonstrated that PET nanofibrils could induce flexible transcrystalline layers around them, leading to greatly improvement of the interfacial adhesion between PET nanofibril and polymer matrix [51]. Figure 13b,c show the Izod impact fracture surfaces of the neat PP foam and PP/PET(F)/POE composite foam, respectively. It can be found that numerous micro cells appeared on the fracture surface of INF composite foam. The PET fibrils resulted in reduced cell size and increased cell density, which probably can prevent crack initiation and propagation by passivating crack tips [52] during the impact tests. Overall, the unique cellular structure combined with enhanced crystallization would promote the slips and fibrillations of polymer chains in impact tests, thereby improving the toughness of foam.

**Figure 14:**
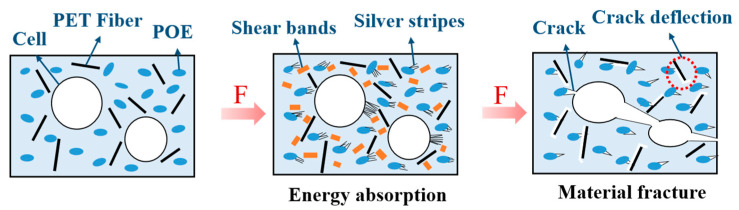
Schematic diagram of the crack toughening mechanism.

It is noteworthy that a large number of PET fibrils and POE additives dispersed uniformly in the frozen layer and cell wall, which is efficient for ductile fracture behavior. Figure 14 illustrated the crack toughening mechanism by cellular structure, PET fibrils and POE additives. Although cells, as well as rubber particle POE, are confirmed frequently to initiate crazes owing to them acting as stress concentrators, both small sized cells and POE particles can control craze growth or craze path. Based on the craze–shear band theory, the dispersed POE particles can induce the silver stripes and shear bands in the PP matrix when subjected to the impact load, and a large amount of impact energy was absorbed. In addition, during the impact testing for most of fiber-reinforced polymeric composite, the crack always propagates along the impact direction, but it will also deflect for an angle from the impact direction. The fibrils may induce a crack deflection perpendicular to the impact direction and transmit the stress to the matrix due to the fiber orientation effect [26]. This will make the matrix participate more actively in the stress transfer, thereby in turn increasing the absorbed energy.

## 4. Conclusions

In summary, a cost-effective and environmentally friendly strategy based on in situ fibrillation and microcellular injection molding process was developed to fabricate lightweight and high toughness PP/PET/POE composite foams. Nanofibrillar PP/PET/POE composite was prepared by combing twin-screw compounding mixed with melt spinning process. Incorporation of POE particles in PP/PET blend played a role of compatibilizer and plasticizer. The average diameter of PET nanofibrils were decreased after introducing POE due to the particle-induced PET domains elaboration. A long and an average diameter of 258 nm were achieved and distributed uniformly in the PP matrix. The rheological analysis data demonstrated that the PET nanofibrils dramatically increased melt viscoelasticity and strength. The isothermal crystallization results showed that PP’s crystal nuclei rate as well as the chain mobility were effectively improved because of the dual heterogeneous nucleation effects of PET nanofibrils and POE particles, and eventually promoted the PP’s crystallization kinetics.

The INF composite foams prepared by MIM achieved a sandwich-like structure, including a solid frozen layer, a shear layer contained stretched cells, and a core layer that consisted of numerous spherical cells. Owing to the improved crystallization and rheological properties, the obvious refined foam structure in the core layer of the PP matrix, with decreased cell size and increased cell density, was obtained by introducing the PET nanofibrils and POE particles. Furthermore, compared with neat PP foams and other melt-blended composites, the PP/PET(F)/POE composite foams exhibited the largest cell deformation in skin layer because the presence of PET nanofibrils increases the resistance to polymer molecular chain motion. Notably, the Izod impact results showed that the PP/PET(F)/POE composite foams exhibited the highest impact strength, increasing by 295.3% compared to PP foams. The existence of oriented PET nanofibrils may induce a crack deflection perpendicular to the impact direction and hinder the crack propagation, thus increasing the absorbed energy. The toughening effect of POE’s uniform dispersion in the PP matrix is another reason for the outstanding impact strength.

The results obtained in this study reveal potential applications for PP based INF composite foams in the manufacturing of lightweight automotive products with enhanced impact properties. Future work would focus on investigating various mechanical and functional properties of INF composite foams, including tensile, flexural and thermal insulation properties.

## Figures and Tables

**Table 1:** Formulations of Neat PP and PP based composites.

Denotation	PET Content (wt.%)	POE Content (wt.%)	Remark
PP	0	0	Neat pellet PP
PP/PET(S)	10	0	Melt blended PP/PET with spherical PET domains
PP/PET(S)/POE	7	3	Melt blended PP/PET/POE with spherical PET domains
PP/PET(F)	10	0	In situ nanofibril PP/PET composite
PP/PET(F)/POE	7	3	In situ nanofibril PP/PET/POE composite

**Table 2:** Summary of Avrami parameters for the isothermal crystallization kinetics of samples.

Samples	*n*	*Z*_t_ (s^−n^)	*t*_1/2_ (s)
PP	2.81	2.48 × 10^−7^	196.84
PP/PET(S)	2.68	4.19 × 10^−5^	37.50
PP/PET(S)/POE	2.51	9.05 × 10^−5^	35.27
PP/PET(F)	2.81	8.27 × 10^−5^	24.91
PP/PET(F)/POE	2.49	4.71 × 10^−4^	18.71

**Table 3:** Statistical results of cell size and cell density at different layers in all foams.

Samples	Core Layer		Shear Layer	
	Cell Size (μm)	Cell Density (cells/cm^3^)	Cell Size (μm)	Cell Density (cells/cm^3^)
PP	100.5 ± 5.3	2.5 × 10^6^	54.6 ± 3.4	6.4 × 10^6^
PP/PET(S)	82.3 ± 4.1	4.4 × 10^6^	42.3 ± 3.1	8.9 × 10^6^
PP/PET(S)/POE	79.6 ± 3.6	4.8 × 10^6^	38.7 ± 2.4	9.5 × 10^6^
PP/PET(F)	64.2 ± 3.9	9.3 × 10^6^	24.8 ± 2.7	3.4 × 10^7^
PP/PET(F)/POE	50.3 ± 2.2	1.6 × 10^7^	18.2 ± 1.6	5.3 × 10^7^

## Data Availability

Not applicable.

## Supplementary Materials

The following supporting information can be downloaded at: https://www.mdpi.com/article/10.3390/polym15010227/s1, Figure S1: SEM micrographs of the cryo-fractured surfaces of: (**a**) neat PP, (**b**) PP/POE blend with 3 wt.% POE and size distribution of POE particles (**c**).
