# Supplementary material for: Lightweight and High Impact Toughness PP/PET/POE Composite Foams Fabricated by In Situ Nanofibrillation and Microcellular Injection Molding
Source: Polymers (Basel). 2023 Jan 1;15(1):227. doi: 10.3390/polym15010227 (PMC9824783; doi:10.3390/polym15010227)
Supplement: Supplementary file 1 [file polymers-15-00227-s001.zip › polymers-2121873-supplementary.pdf]

Supporting Information

Lightweight and High Impact Toughness PP/PET/POE Composite Foams Fabricated by In Situ Nanofibrillation and Microcellular Injection Molding

Junwei Sun [1], Qian Li [1,*], Yufan Jiang [2], Jing Jiang [3,*], Lian Yang [3], Caiyi Jia [3], Feng Chen [4] and Xiaofeng Wang [1]

[1] School of Mechanics and Safety Engineering, Zhengzhou University, National Center for International Research of Micro-Nano Molding Technology, Zhengzhou 450001, China
[2] School of Materials Science & Engineering, Zhengzhou University, Zhengzhou 450001, China
[3] School of Mechanical & Power Engineering, Zhengzhou University, Zhengzhou 450001, China
[4] College of Material Science and Engineering, Zhejiang University of Technology, Hangzhou 310014, China
* Correspondence: qianli@zzu.edu.cn (Q.L.); jiangjing@zzu.edu.cn (J.J.); Tel.: +86-15617960455 (J.J.)

S1. The POE distribution in composites

To investigate the morphologies of POE in the PP/PET composites, we prepared PP/3wt%POE blend which served as control group, and its cryogenic fractured surface is shown in Figure S1(b). It can be seen from the Figure S1(b) that the distribution of POE in the PP matrix was relatively uniform, and the 'sea-island' structure was formed in the PP matrix, which was mainly because the compatibility of POE and PP was better. It can also be observed that the average cell diameter of POE particles was so small, reaching 66nm, as shown in Figure S1(c).

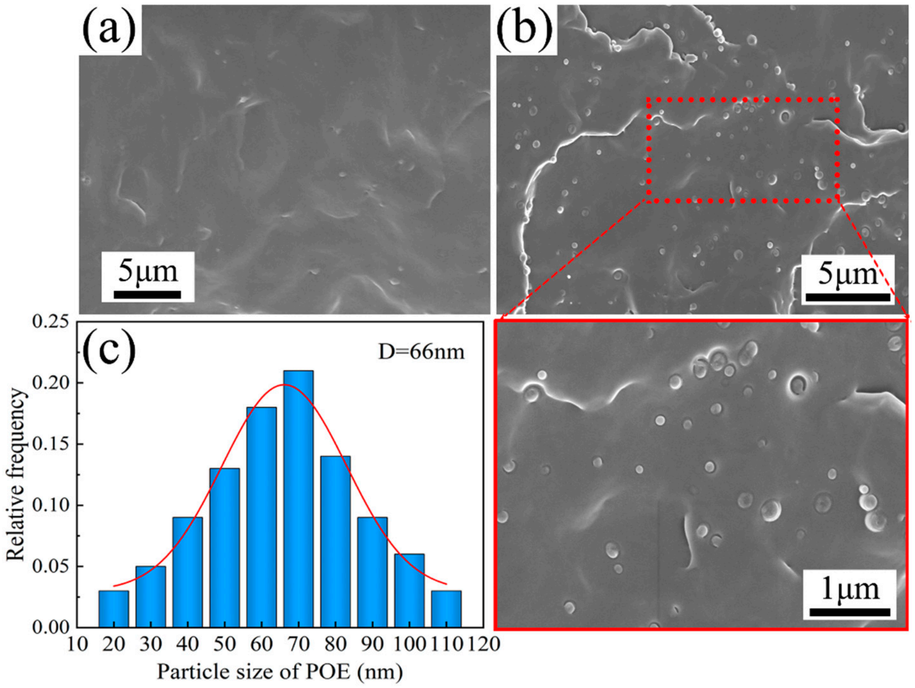

Figure S1. SEM micrographs of the cryo-fractured surfaces of: (**a**) neat PP, (**b**) PP/POE blend with 3wt% POE and size distribution of POE particles (**c**).
